# High-Performance Liquid Chromatography Method for the Determination of Folic Acid in Fortified Food Products

**DOI:** 10.1080/15376510701623870

**Published:** 2008-06-23

**Authors:** A. Lebiedzińska, M. Da̧browska, P. Szefer, M. Marszałł

**Affiliations:** Department of Food Sciences, Medical University of Gdańsk, Al. Gen. J. Hallera 107, Gdańsk 80-416, Poland; Department of Toxicology, Medical University of Gdańsk, Al. Gen. J. Hallera 107, Gdańsk 80-416, Poland

**Keywords:** Cereal-Grain Products, Folic Acid, Fortified Fruit Juices, HPLC-ECD Analysis

## Abstract

Reversed-phase high-performance liquid chromatography, coupled with coulometric electrochemical detection, was successfully applied for the quantification of added folic acid (FA) in fortified fruit juices and cereal products. The method allowed good separation of the 5-HCO-H_4_ folate and folic acid in cereal samples. The retention times of vitamins were repeatedly determined by isocratic elution using 40 mM sodium phosphate dibasic, heptahydrate buffer, and 8% acetonitrile (v/v) (0.9 mL/min, pH 5.5) as mobile phase with the Supelco LC 18 column 5 μm (25 cm × 4.6 mm). Folate concentrations were measured using a trienzyme (hog kidney folate conjugase, α-amylase, and protease) folate extraction method.

Folate is a generic term referring to the mono- to polyglutamate derivatives of pteroic acid that occur naturally in many foods, and have a closely related biological activity. During the last decade, the importance of adequate folate intake has become well recognized in the reduction of the rate of neural tube defects. These vitamins cofactors are essential for the synthesis of purines and pyrimidines and in the production of methionine from homocysteine (Ball 2006; Bailey 2005; Cravo and Camilo 2000; Das 2003; Finglas et al. 2006; Malinow et al. 1999). The most important dietary sources are fortified foods, liver, green vegetables, and cereal products (Ball 2006). Naturally occurring folates in reduced form are heat labile and readily destroyed by oxidation. Fortification of food with vitamins is intended to compensate for the loss of these compounds due to the heat treatment to which they are subjected during manufacture (Akhtar et al. 1999). The predominant natural forms of folate in animal products are 5-methyltetrahydrofolate (5-CH_3_–H_4_ folate) and tetrahydrofolate (H_4_ folate); in fruits and vegetables 5-CH_3_–H_4_ folate; and in cereal products 5-formyltetrahydrofolate (5-HCO-H_4_ folate) and 10-formyl-folic acid, and they have been detected in reasonable amounts, together with 5-CH_3_–H_4_ folate and folic acid (Ball 2006).

The determination of B vitamins in various samples is rather difficult due to the chemical instability and complexity of the matrices in which they usually exist. Vitamins are most often determined in the free form, which involves hydrolysis of the phosphorylated forms and/or those bound to proteins (and optionally glycosylated) during the extraction step performed prior to the chromatographic separation. The extraction procedure was considered to have the greatest impact on analytical results. During the last decade, the use of a trienzyme treatment method has been developed for more efficient extraction of folates from food than the conventional methods. This method includes the use of protease and amylase in combination with traditional folate conjugase treatment following heat extraction (De Souza and Eitenmiller 1990; Hyun and Tamura 2005; Johnston et al. 2002; Tamura 1998; Yon and Hyun 2003). The methods for vitamin determination require the individual approach to analyze each vitamin by different physical, chemical, and microbiological analytical procedures (Eitenmiller and Landen 1999). Usually the folate content of food is quantified by microbiological assays, carried out as *Lactobacillus casei*-based turbidimetric assay or titrimetric methods (AOAC International 2003). To the most popular chromatographic techniques belong high-performance liquid chromatography (HPLC). The modifications of the HPLC coupled to ultraviolet-visible absorbance (Heudi et al. 2005), fluorometric (Gregory et al. 1984: Gujska and Kuncewicz 2005; Ruggeri et al. 1999), and electrochemical detections have been presented in the literature. The gradient elution is usually applied for the separation of individual folate determination (Eitenmiller and Landen 1999). Their determination by ion-pairing chromatography with reversed phase is the most frequent method (Breithaupt 2001; Eitenmiller and Landen 1990; Pfeiffer et al. 1997). Alternative methods are based on redox reaction with electrochemical detectors such as coulochemic instruments (Bagley and Selhub 2000; Flangan et al. 2005). The B vitamins are easily oxidized or reduced with online working electrode in electrochemical cell. The high sensitivity and lower limits of detection are obtained using the electrochemical detection allowing the analysis of folate from distribution in red blood cells and lymphocytes (Bagley and Selhub 2000).

This work presents the application of several procedures including a trienzyme extraction and reversed-phase HPLC with coulometric electrochemical detection for the quantification of folic acid and 5-HCO-H_4_ folate in fortified food products.

## MATERIALS AND METHODS

All solutions were prepared with analytical reagent grade compounds. Sodium phosphate dibasic heptahydrate was obtained from Sigma-Aldrich (St. Louis, MO, USA). Other reagents were also of HPLC grade–that is, orthophosphoric acid (Riedel de Häen, Seelze, DA), and solvents: acetonitrile and water (Baker, HPLC Analyzed). Water was purified using the Milli-Q system from Millipore (Eschborn, Germany). Enzymes, α-amylase, protease, and kidney acetone powder, porcine, type II were provided by Sigma Chemical (Sigma Aldrich). The hog kidney conjugase was prepared according to Gregory at al. (1984) and Pfeiffer et al. (1997). The folate standards (folic acid and 5-formyltetrahydrofolate [calcium salt]) were obtained from Sigma Aldrich. The certified reference material wholemeal flour (CRM 121) was obtained from the Community Bureau of Reference, BCR (Belgium). The reference material was subsampled (5 g) and stored frozen.

Fruit juices, fruit drinks (vitamin fortified), and cereal products were purchased from local supermarkets. All samples were commercially available. The various solid products were finely ground, subsampled (50–100 g), and stored at −18°C until analysis.

The various samples of fruit juices (20 mL) were finely mixed with additional 20 mL phosphoric buffer (pH 6.8); cereal products (2 g) were homogenized by stirring in 20 mL of phosphoric buffer (pH 6.8) and left for 10 min in a water bath at 100°C. After cooling the pH was adjusted to 4.9. The enzymatic digestion prior to separation and quantification step made it possible to release the vitamins bound to proteins or sugars. The extraction procedure was based on a study of Gujska and Kuncewicz (2005). The homogenate was subjected to trienzyme treatment: 3 mL hog kidney folate conjugase and 1 mL α–amylase (4 h at 37°C) followed by 2 mL protease (1 h at 37°C). After enzyme incubation, samples were heated to 100°C for 5 min to inactivate the enzyme, then cooled in ice and centrifuged. The residue was resuspended in extraction buffer and centrifuged again, and the combined supernatants were filled to an exact volume (50 mL), filtered through a Whatman 1 Chr filter paper, then flushed with nitrogen and stored at −30°C. Samples were prepared under dim light and then kept in the dark. Prior to HPLC analysis all samples were filtered through a 0.22-μm pore-size filter. Samples should always be protected from light and working under subdued lighting conditions. The working calibration solution was prepared daily and further diluted with mobile phase.

The HPLC system was coupled with pump P 580 (Dionex) and Coulochem II electrochemical detector (ESA, Inc.) operated by the Chromeleon Chromatography Management System.

## HPLC ANALYSIS

The samples were separated isocratically on the reversed-phase LC 18 column 5 μm (25 cm × 4.6 mm), produced by Supelco, Inc. The mobile phase, consisting of 40 mM sodium phosphate dibasic, heptahydrate, and 8% acetonitrile (v/v), was adjusted to pH 5.5 with 85% phosphoric acid. The mobile phase was filtered through a 0.22-μm pore membrane filter and degassed before use. The column was equilibrated at 25°C at a flow rate of 0.9 mL/min. The volume of injection was 20 μL. The column eluate was monitored with electrochemical detector Coulochem II (model 5020A, ESA) equipped with dual analytical cell (model 5010) and guard cell (model 5020). The guard cell was connected in-line before injection port and was used to eliminate the interference with baseline stability. The detector response was set to give a full-scale detection for 1 μA and 50 μA current output received from the analytical cell. The HPLC was operated in a constant flow mode and the flow rate was kept at 0.9 mL/min. Peak area of the electrochemical signal at the porous graphite electrode was used for the analysis of folate form distribution. In the EC detection both sensitivity and selectivity were adjusted by varying the potential maintained between the working and reference electrodes; higher potentials induce a response from more compounds and therefore compromise selectivity. Potential is related to the free energy of the overall reaction. The current of the measurement cell is correlated with the working electrode potential. The relation between potential and resulting current is plotted in a current voltage diagram (Flangan et al. 2005). Optimum working potential can be ascertained by repeated injections of a solution of the analyte at differing detector voltages. For obtaining optimum detection, the electrode potentials for the guard cell, electrode E1, and electrode E2 were set at 0.85 V, 0.20 V, and 0.80 V, respectively. A high enough potential at the electrodes has to be applied in order to achieve high sensitivity and baseline stability.

Electroactive compounds, such as folate, begin to oxidize at a specific electrode, depending on their individual oxidation potential. Because coulometric electrodes operate at close to 100% efficiency, compounds that oxidize at lower potential electrodes are completely oxidized; therefore, the oxidized compounds are not detected at higher potentials (Flangan et al. 2005). The electroactivity of compounds monitored with a coulometric detector is dependent on the presence of functional group of molecules. The current peak at the positive potential is directly related to the concentration of the analyzed vitamins showing that the response should be caused by the electroactivity of vitamins.

## RESULTS AND DISCUSSION

The calibration concentration range was established through consideration of the interval of values attributable to natural concentrations specific for each of the food samples studied. Such approach was necessary to give accurate, precise, and linear results. The calibration graphs were constructed by plotting the peak area based on results corresponding to triplicate injections of vitamins standards. The linearity of the calibration graphs was highly acceptable as proved based on a high correlation coefficient (*r* ≥ 0.998). The concentration ranges for food samples were as follows: 50 to 2500 and 50–5000 μg/mL for folic acid and 5-HCO-H_4_ folate, respectively. The limit of detection (LOD) of the method was used, calculated as three standard deviations of the background noise of the standard finally diluted in the same buffer as the food sample. The LOD for standard solution of folic acid amounted to 1.3 ng/mL. Subsequently, the LOD established for standards of 5-HCO-H_4_ folate was 4.5 ng/mL.

**FIGURE 1:**
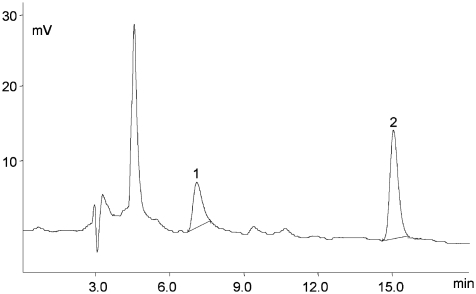
Chromatogram of the main folate forms present in wheat flour type 550 1—5-HCO-H_4_ folate (T_R_ 7.0 min, 0.85 μg/mL), 2—folic acid (*t*_R_ 15.0 min, 1.13 μg/mL). EC detector sensitivity 1 μA. LC 18 column 5 μm (4.6 mm × 25 cm) and a mobile phase, consisting of 40 mM sodium phosphate dibasic, heptahydrate buffer, and 8% acetonitrile (v/v), pH 5.5. The sample size and the final dilution used for the analyzed vitamin determination varied according to the vitamin content in products.

**FIGURE 2:**
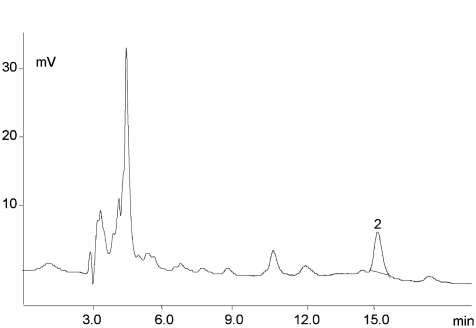
Chromatogram of the folic acid in fortified fruit juices—2 folic acid (*t*_R_ 15.0 min, 3.2 μg/mL). EC detector sensitivity 1 μA. LC 18 column 5 μm (4.6 mm × 25 cm) and a mobile phase, consisting of 40 mM sodium phosphate dibasic, heptahydrate buffer, and 8% acetonitrile (v/v), pH 5.5. The sample size and the final dilution used for the analyzed vitamin determination varied according to the vitamin content in products.

Figures 1 and 2 illustrate chromatograms of the vitamins studied the analyzed food samples. The calibration concentration range was established through consideration of the interval of values attributable to natural concentrations specific for each of the food samples studied. The retention time (10 replicates) for folic acid was T_R_ 15.0 ± 0.3 min, while its value for 5-HCO-H_4_ folate was T_R_ 7.0 ± 0.2 min. A certified reference material as wholemeal flour (CRM 121) was analyzed to check the accuracy of the analysis. The analytical results obtained for the analyzed certified reference material (as folic acid) were satisfying–that is, the recoveries (as a measure of accuracy) for the analytes studied ranged between 98.0% and 103.6%.

Table 1 summarizes the results obtained for the determination of folic acid in the analyzed fruit drinks and fruit juices. The levels of folic acid detected by the present method ranged from 58% to 116% compared with the amounts labeled. Only in one case the folic acid concentration was higher than labeled. Consequently, the corresponding serving size of juice samples does not provide the amounts of vitamin expected by the consumers. These mean values were noticeably lower than FA values measured by Breithaupt (2001). The folate contents in some commercial cereal-grain products are presented in Table 2. For five cereal-grain products, mean analyzed values ranged from 154.3 to 414.9 μg/100 g^−1^ of the products and were significantly higher than label statements. Our data indicate that the folate content in some enriched products is slightly higher than expected from the label declaration. Data reported by Rader et al. (1998, 2000) for total folate content of enriched cereal-grain products showed that some products were fortified at “higher-than-minimum” levels. The potential for excesses of folic acid is of concern because this vitamin is the type for which a tolerable upper intake level has been recommended (Institute of Medicine 1998).

**TABLE 1:** Determination of folic acid in commercial vitamin-fortified fruit juices and fruit drinks (μg/100 mL)

Fruit juice samples	n	Amount labeled	Amount determined^a^
Fruit juices—multivitamin	9	30	21.38 ± 0.99 (70%) (19.81–24.09)
Fruit nectar—multivitamin	16	30	35.21 ± 0.78 (116%) (34.39–36.10)
Fruit drink (apple, mango, pears)	9	30	17.43 ± 0.29 (58%) (17.22–17.63)
Fruit juice (orange, 100%)	9	30	19.11 ± 1.51 (64%) (17.00–21.24)
Fruit juice (orange, banana)	9	30	27.52 ± 0.96 (92%) (26.54–28.5)

a Data are averages of five determinations.

n = number of samples.

**Table 2:** Determination of folic acid in commercial vitamin-fortified cereal-grain products (μg/100 g)

Product	n	5-HCO-H_4_^a^	Folic acid^a^	Total (as folic acid)	Amount labeled
Wheat flour type 450	3	ND	154.3 ± 1.6 (153.2–155.0)	154.3	100
Wheat flour type 550	3	68.5 ± 3.7 (66.0–72.7)	283.7 ± 1.3 (282.6–284.6)	352.2	100
Toasted oat flakes	3	ND	95.7 ± 1.1 (93.9–96.9)	95.7	Unfortified
Corn flakes	3	29.0 ± 3.8 (23.0–30.6)	76.6 ± 1.6 (75.2–78.3)	105.6	Unfortified
Corn flakes—fitness	3	98.8 ± 3.9 (94.6–102.1)	174.8 ± 2.9 (165.1–170.2)	273.6	340
Corn puffs—cinnamon	3	67.6 ± 4.7 (63.4–72.7)	318.8 ± 2.3 (316.9–321.3)	386.4	170
Fruit flakes	3	153.8 ± 4.6 (148.5–156.9)	261.1 ± 12.4 (246.8–269.2)	414.9	260
Fruit pops	3	128.3 ± 3.4 (124.9–131.6)	161.7 ± 0.9 (161.0–162.7)	290.0	170
Macaroni with spinach	3	33.7 ± 9.2 (26.4–44.0)	194.8 ± 7.2 (187.9–202.2)	228.5	Unfortified

a Data are averages of five determinations.

n = number of food samples.

## CONCLUSIONS

The method used in this study (trienzyme deconjugation, extraction procedure, reversed-phase HPLC separation) provides sufficient selectivity to resolve folic acid from other compounds in fortified fruit juices and cereal products and allowed a good separation of the 5-HCO-H_4_ folate in cereal samples. Coupling this separation with coulometric detection provides a suitable instrumental method for determining folic acid in food samples. The simple mobile phase and the isocratic elution used to separate folic acid and folate yielded low detection limits, good sensitivity, and resolution within a minimum analysis time of 16 min. The simplicity of the procedure, satisfying detection and sensitivity (coulochemic electrochemical), should make our method possible to adopt for routine quality control of folic acid-enriched foods.
